# Overcoming velocity broadening effects in magnetic beam encoded microscopy: a wavelength-resolved imaging scheme

**DOI:** 10.1098/rsos.251238

**Published:** 2025-10-22

**Authors:** Morgan Lowe, Elena Cates, Helen Chadwick, Gil Alexandrowicz

**Affiliations:** ^1^ Department of Chemistry, Swansea University, Swansea, UK

**Keywords:** atomic and molecular beams, neutral beam microscopy, magnetic resonance imaging

## Abstract

The recently developed magnetic encoding beam microscopy approach to imaging with neutral atomic beams has a spatial resolution that is limited by the spread of velocities within the beam. Here we present a solution for overcoming this restriction which is based on adding a homogeneous magnetic field and resolving both the spatial resolution and the de Broglie wavelength of the particles using sequential Fourier transforms. Numerical simulations are used to demonstrate the enhanced resolution obtained with this approach, and how features which were lost in the spatial reconstruction due to the spread of velocities can be recovered. Wavelength/velocity resolved profiles that were reconstructed from experimental data are presented, demonstrating how the scheme can be applied in practice.

## 1. Introduction

Magnetic beam encoding microscopy (MBEM) [1] is a recent development of neutral helium microscopy [2], where instead of using pin holes [3,4] or focusing elements such as Fresnel zone plates [5,6] to obtain spatial resolution, magnetic field gradients are used to manipulate the magnetic moment of the beam particles, encode their position in space and allow a reconstruction of the object which the beam interacts with. The MBEM approach has various advantages and disadvantages when compared with established techniques for neutral beam microscopy. For example, improving the resolution in MBEM should scale much more favourably with the signal intensity, and correspondingly also the measurement time, when compared with pin-hole techniques [1]. On the other hand, the MBEM requires a much more complex and larger set-up than a pin-hole microscope [7], and has so far only been demonstrated in a one-dimensional (1D) transmission proof-of-principle study. One significant hurdle with improving the resolution of MBEM is that the encoding procedure is inherently velocity dependent and the distribution of velocities within the particle beam leads to a broadening (blurring) of the resolution which can be obtained [1].

In this paper, we introduce a method for circumventing the velocity broadening effect. In §2, we briefly describe the basic principles of MBEM and the experimental set-up which can perform a 1D profiling experiment. In §3, we explain and show, using numerical simulations, the effect of velocity spread on the resolution. In §4, we present the idea of wavelength resolved imaging, and show numerically that high resolution reconstruction can be obtained even when the beam has a wide velocity spread. In §5, we present an experimental demonstration of wavelength resolved spatial reconstruction, which reaffirms the results obtained by the numerical simulation. In §6, we summarize the main findings of this work.

## 2. Basic spatial encoding principle of MBEM

**Figure 1.**
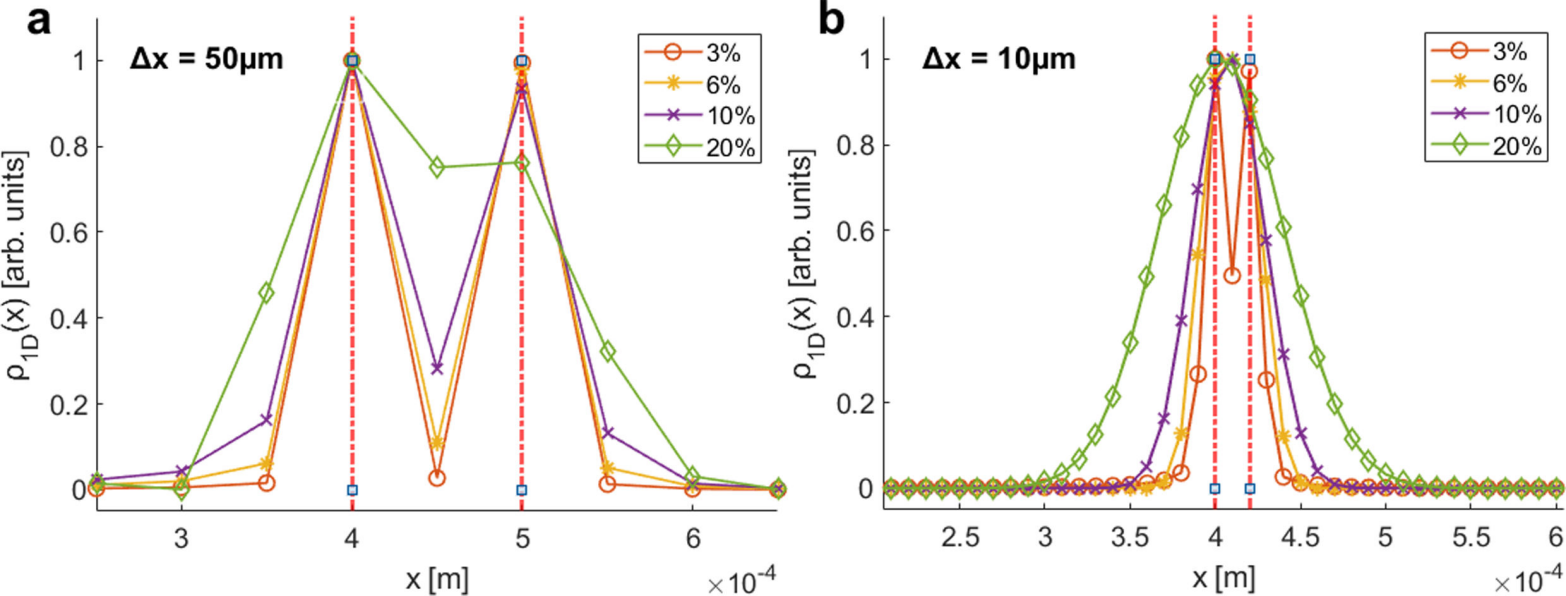
Spatial reconstructions of two simulated sharp features, positioned at approximately 0.4 mm and separated by 1 pixel (spatial bin), for two different resolutions: (a) 50 µm and (b) 10 µm. The reconstructions are shown for Gaussian velocity distributions with full-width half-maximum (FWHM) values of 3%, 6%, 10% and 20%, illustrating the impact of velocity spread.

A detailed explanation of the encoding principle of MBEM has been recently published [1], and is illustrated graphically in fig. 1 and in the videos of the original publication’s electronic supplementary material. Below we briefly repeat the main points. MBEM leverages the magnetic moment response to external magnetic fields as a means of encoding the lateral positions of the beam particles perpendicular to the beam propagation direction, it is an atomic beam analogue of phase encoding magnetic resonance imaging [8,9]. The experiment starts with creating a spin polarized atomic beam, where the axis along which the beam propagates is denoted as $\hat{z}$, and direction along which the spins are initially polarized along is denoted as $\hat{x}$. The beam, which is characterized by a mean velocity, $\bar{v}$, is passed through a magnetic field gradient device with a length of L. In the first MBEM demonstration [1] and also in the experimental work presented here, the encoding device produces a magnetic field in the $\hat{y}$ direction which changes as a function of the x coordinate, i.e. $\frac{dB_{y}}{dx}$. Note that we are following the axes convention used in previous work [1], i.e. that both the direction of the encoding magnetic field ($\hat{y}$) and the direction of the gradient ($\hat{x})$ are both perpendicular to the atomic beam propagation axis ($\hat{z}$). When the beam particles pass through the encoding device the magnetic moments precess in the xz plane, with a frequency which depends on the magnitude of the $B_{y}$ field they encounter. As the beam passes through the encoding device, each beam particle accumulates a classical spin-phase $\phi =\gamma \frac{dB_{y}}{dx}x\frac{L}{\bar{v}}$, which is proportional to its spatial position within the device, x, the time spent in the field, $t=\frac{L}{\bar{v}}$, the strength of the gradient applied, $\frac{dB_{y}}{dx}$, and, γ, the gyromagnetic ratio of ^3^He.

After passing through the encoding device, the beam interacts with the sample and the remaining beam is either transmitted towards a detector in transmission-mode, or reflected from the sample surface towards the detector in reflection-mode. The interaction of the beam with the sample can take place either before or after passing through the encoding device. The fraction of the beam which continues after the interaction with the sample passes through a spin selector (we use a magnetic hexapole for this), before reaching the particle detector. The detector signal is measured over a range of gradient strengths, $S\left(k_{x}\right)$, where $k_{x}=\frac{1}{2\pi }\gamma \frac{L}{\bar{v}}\frac{dB_{y}}{dx}$. Following detection, it is possible to reconstruct the 1D profile of the beam through the Fourier relation between the complex signal, $S\left(k_{x}\right)$, and the 1D spatial profile of the beam, $\rho_{1D}\left(x\right)$. The resolution within which the profile is reconstructed is related to how far in reciprocal $k_{x}$ space we can measure, and consequently is related to the maximum strength of gradient field we can use. By contrast, the field-of-view (FOV), or the range of the profile reconstruction in a 1D experiment, is determined by the increments of gradient strength applied, and must be set such that no beam particles are situated beyond the FOV as to not produce signal aliasing and artefacts in the reconstructed profile. The principle explained above can be expanded from 1D profiles to two-dimensional (2D) imaging using a second encoding device as was demonstrated using numerical simulations [1].

## 3. Velocity spread broadening

While the resolution of a MBEM experiment can be improved by increasing the strength of the magnetic field gradient or its length, the resolution that can be obtained will be restricted by velocity broadening effects, which are created by the spread of velocities within the ^3^He beam [1]. The effect of the velocity is easy to understand if we rewrite the expression for the accumulated Larmor phase, replacing the average beam velocity with the individual velocity of each particle in the beam, v, i.e. $\phi \left(x,v\right)=\gamma \frac{dB_{y}}{dx}x\frac{L}{v}$, i.e. faster beam atoms will spend a shorter period of time within the gradient field and therefore will accumulate a phase that would be indistinguishable from slower atoms at spatial positions closer to the centre of the beam, and vice versa. Another important point which is made obvious by looking at the equation for ϕ(x,v), is that the effect of velocity spread on the accumulated Larmor phase and correspondingly on our reconstructed profile/image, will become more problematic as the resolution of the experiment is enhanced (stronger gradient, or longer encoder) as well as for particles which are further away from the zero-field region at the centre of our encoding device (i.e. further away from x=0).

To illustrate the limitations in achieving high-quality and high-resolution images with MBEM in the presence of velocity-spread-related broadening, we performed numerical simulations. Similarly to those presented in our previous work [1], they were based on solving the dynamics of the magnetic moment of particles as they move through the magnetic fields of the simulated set-up. The incident beam was simulated as a beam of spin-½ ^3^He atoms, moving at an average velocity of 756 ms^−1^, with a Gaussian distribution of velocities, the width of which was varied. For the spatial distribution of the post sample interaction beam, we chose two delta functions separated by only one point (i.e. the distance between the peak positions is twice the lateral resolution of the reconstruction, 2Δx). In this case the degree of velocity related broadening can be assessed by the ability to separate the two features, i.e. to reconstruct a dip in intensity between the two peaks. Using spatial distributions consisting of two sharp close-by peaks, has been shown as a useful way of quantifying resolution of neutral beam microscopes [10].

Panels a and b in figure 1 correspond to reconstruction resolutions which are equal to 50 μm and 10 μm correspondingly. The delta peak spatial distributions of the beam we are simulating are shown by the two vertical red dashed lines, while the calculated reconstructions for beams with different velocity distribution widths are shown by the different markers and curves denoted in the legend alongside the value of the full width half maximum of their velocity distributions.

As the width of the velocity distributions is increased the reconstructed peaks broaden and their intensity smears into the adjacent pixels, as a result the two peaks merge and eventually the dip in between can no longer be observed. It can be noticed that in figure 1a, at an FWHM of 10% and a resolution of 50 μm, it is still possible to distinguish between both sharp features (for this given spatial position), however when enhancing the resolution of the scan by a factor of 5, as in figure 1b, the effect of the broadening is quite dramatic. For this resolution, the only case where a dip can still be observed is for a beam with an FWHM of 3%, whereas for any wider velocity distribution the two peaks merge into one in the reconstructed profile. This emphasizes the point we mentioned earlier, i.e. that the effect of the velocity broadening becomes more important for higher resolutions.

## 4. Achieving high resolution in the presence of a broad velocity spread

Our approach to solve the velocity broadening problem is based on incorporating wavelength reconstruction into the imaging scheme. We start by expressing the distribution of different velocities in our beam as a distribution of de Broglie wavelengths $\rho^{\prime }\left(\lambda \right)$ where $\lambda =\frac{h}{mv}$*,* where h,m and $v^{\prime }$ are Planck’s constant, the mass and the velocity of the ^3^He particles, respectively. The motivation for using wavelengths instead of velocities is that this simplifies the mathematics and leads to a Fourier transform relation between the distribution of wavelengths within the beam, and the signal measured in a spin echo experiment [11–14]. When a ^3^He particle travels through a homogeneous magnetic field, which we denote, $B_{1}$, it undergoes Larmor precession, and the classical spin phase it accumulates can be written as $\phi =\gamma \frac{m\lambda }{h}B_{1}L_{1}$, where γ is the gyromagnetic ratio of ^3^He [13,14], $B_{1}$ and $L_{1}$ describe the amplitude and length of the homogeneous magnetic field (for a field which varies in amplitude along its length, the product $B_{1}L_{1}$ is replaced by the path integral of the field [13,14]). The phase can be expressed more compactly as $\phi =2\pi k_{1}\lambda$ where we define a new reciprocal space vector, $k_{1}=\frac{\gamma mB_{1}L_{1}}{2\pi h}$. Similarly to the derivation of the imaging principle of MBEM [1], we can now define a complex signal, $S\left(k_{1}\right)$, where the real and imaginary parts are the components of the total beam magnetization along two perpendicular axes, and show that it is related by a Fourier transform to the distribution of wavelengths in the beam, $\rho^{\prime }\left(\lambda \right)$ [13,14]


(4.1) $$S\left(k_{1}\right)\propto \int_{-\infty }^{\infty }\rho^{\prime }\left(\lambda \right)e^{2\pi ik_{1}\lambda }d\lambda .$$

The relation expressed in equation (4.1) means $\rho^{\prime }\left(\lambda \right)$ can be reconstructed from the signal using an inverse Fourier transform. We note that equation (4.1), is written as an integral over an infinite range following the usual definition of a Fourier transform relation. In practice, the signal is a discrete sum over the finite number of particles in a beam. The large number of beam particles contributing to each measurement and the fact that $\rho^{\prime }\left(\lambda \right)$ vanishes for λ which are not populated in the beam justifies the use of an integral over an infinite range.

Experimentally, $k_{1}$ is varied by adjusting the strength of the homogeneous magnetic field, $B_{1}$. As with the image reconstruction formalism, discrete Fourier transform conditions again apply; the increments in field strength determining $\Delta k_{1}$ must be small enough to satisfy the Nyquist–Shannon sampling criteria, ensuring no aliasing of the signal and thus correspond to the slowest atoms present in the beam (largest wavelengths), given by $\lambda_{max}=\frac{1}{2\Delta k_{1}}$.

With both Fourier schemes now established we can unify the generation of 1D spatial reconstructions with wavelength resolution and later express how a spatial image for each reconstructed wavelength, $\rho_{\lambda }\left(x\right)$, can be generated. When a particle passes through a homogeneous field first and then through a gradient field it accumulates phase contributions of $k_{1}\lambda$ and $k_{x}x$ correspondingly. However, as we mentioned earlier $k_{x}$ is velocity dependent. Using the de Broglie wavelength to express this dependency instead of velocity, we can write $k_{x}=\frac{1}{2\pi }\gamma \frac{m\lambda L}{h}\frac{dB_{y}}{dx}$. The signal after passing both fields, and integrating over all the wavelengths and positions in the beam can be written in a rather compact form,


(4.2) $$S\left(k_{1},k_{x}\right)=\iint_{-\infty }^{\infty }\rho^{\prime }\left(\lambda \right)\rho \left(x\right)e^{2\pi ik_{x}x}e^{2\pi ik_{1}\lambda }dxd\lambda .$$

Equation (4.2) resembles the standard form of a 2D Fourier transform between $S\left(k_{1},k_{x}\right)$ and the product $\rho^{\prime }\left(\lambda \right)\rho \left(x\right)$, with the exception that the phase of the first complex exponent also depends on λ through $k_{x}$. In appendix A, we show that by performing a 1D Fourier transformation in $k_{1}$, choosing a specific wavelength value, $\lambda^{\prime }$, and then performing a second 1D Fourier transformation we can reconstruct wavelength resolved profiles $\rho_{\lambda^{\prime }}\left(x\right)$. Each of these wavelength resolved profiles correspond to a narrower bandwidth in wavelength, Δλ, which significantly reduces the velocity broadening effects. Finally, one other effect which needs to be considered when creating wavelength resolved profiles, is that both the range and the intervals of $k_{x}$ are dependent on λ, and correspondingly so are the resolution and field of view of different wavelength resolved profiles.

**Figure 2.**
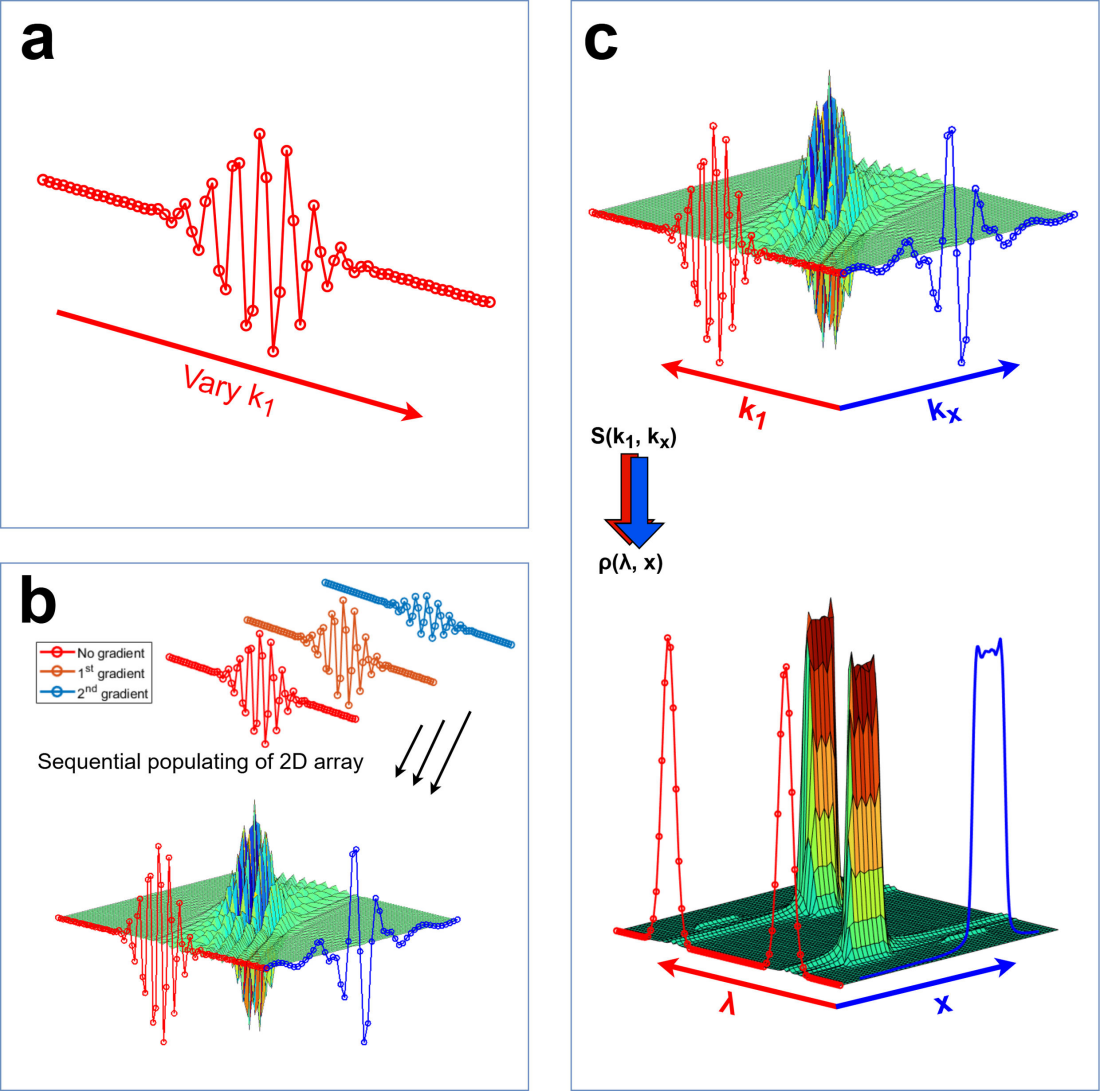
Schematic outline of the process for generating velocity resolved images. Steps (a) and (b) illustrate the workflow for generating the 2D signal *S*(*k*_1_,*k*_*x*_), while step (c) illustrates the post-processing analysis which involves performing Fourier transforms along *k*_1_ and then along *k*_*x*_.

Figure 2 illustrates the process of measuring wavelength resolved spatial profiles. Panel (a) illustrates the signal acquired by scanning $B_{1}$, and correspondingly $k_{1}$, in small intervals around the spin echo point while keeping the gradient field initially turned off ($k_{x}=0$).

The current sweep performed in $B_{1}$ is repeated while incrementally increasing the applied gradient field strength to simultaneously span $k_{x}$-space, as shown in panel (b). This process continues until the array $S\left(k_{1},k_{x}\right)$ is fully populated; achieved by performing a measurement of the collective beam polarization in $\hat{x}$ for all combinations of $k_{1}$ and $k_{x}$ (i.e. from -$k_{1,max}$ to $k_{1,max}$ in steps of $\Delta k_{1}$ and from -$k_{x,max}$ to $k_{x,max}$ in steps of $\Delta k_{x}$).

Panel (c), illustrates the two Fourier transformations which are performed on the 2D signal $S\left(k_{1},k_{x}\right)$. Looking at the results that are obtained after Fourier transforming along both dimensions (the lower plot), we see that the signal is concentrated in two regions in wavelength space, one of these corresponds to wavelength values which are actually populated in the beam, and the other is a mirror peak appearing at negative wavelength values. The mirror peak, contains no further information and can be ignored. It appears when we only measure (or simulate) one spin projection, which essentially converts the Fourier transform in equation (4.1) into a Cosine transform, the magnitude of which is always symmetric. Another point which becomes apparent when looking at panel (c), is that there is no point performing the second Fourier transform for wavelengths which do not have significant intensity (i.e. wavelengths which are not significantly populated in the beam).

Once wavelength resolved profiles are reconstructed for wavelength values with significant intensity, they can either be used separately or their profiles can be combined. In both cases they enhance the ability to resolve image detail which otherwise would be obscured by velocity-related broadening, as we will show below using results from numerical simulations.

**Figure 3.**
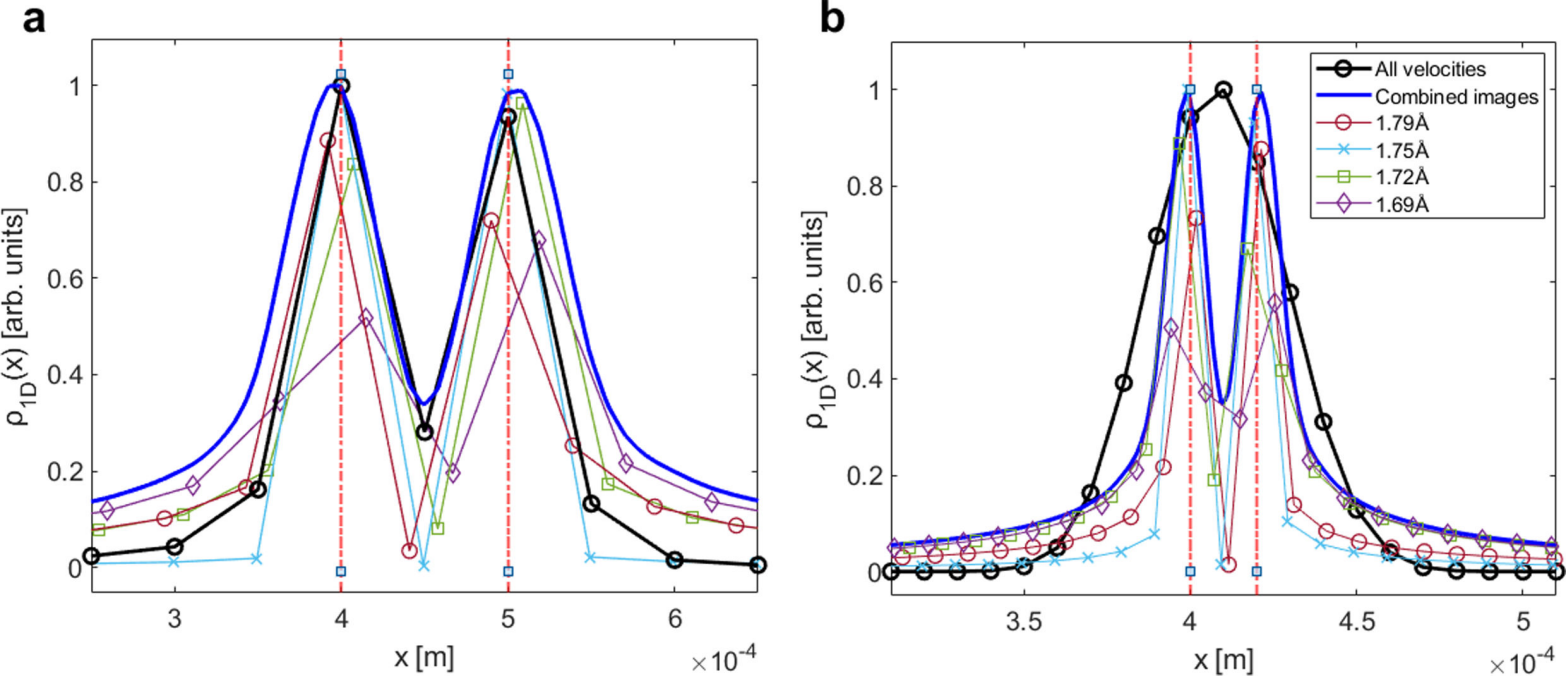
Simulated spatial reconstructions of two sharp image features separated by 1 pixel and positioned around 0.4 mm. The simulations were performed for 201 echo measurement points, and for two different resolutions: (a) 50 µm and (b) 10 µm. The FWHM of the simulated gaussian velocity distribution was 10%. Plots in black outline the image produced with all velocities included (i.e. without performing this technique extension) while the blue line shows the summative contributions of all wavelength-resolved images interpolated and combined into a single image.

Figure 3 shows numerical simulations of wavelength resolved profiles. To obtain these profiles we first calculated a signal using the numerical simulation which was used to demonstrate the velocity broadening effect in figure 1, with the difference that the calculation included scanning both the homogeneous field value ($B_{1}$) and the field gradient value, i.e. calculating the 2D signal matrix $S\left(k_{1},k_{x}\right)$. The simulated beam had a mean velocity of $v=756\;\text{m}{\text{s}}^{\text{−1}}$ and a velocity spread with an FWHM of 10%. The signal was then processed using the procedure described above to produce wavelength resolved spatial profiles. The reconstructed profiles shown in panels a and b were calculated for the same conditions used to generate the data in figure 1, i.e. reconstruction resolutions of 50 μm and 10 μm correspondingly, and a spatial profile which consists of two delta functions separated by only one point (i.e. separated by twice the lateral resolution of the reconstruction, 2Δx). The delta function spatial distributions are shown by the vertical dashed (red) lines.

The full lines show wavelength resolved profiles extracted using the procedure described above for de Broglie wavelengths of 1.69 Å, 1.72 Å, 1.75 Å and 1.79 Å, which correspond to wavelengths which were significantly populated in the simulated beam. All of the wavelength resolved profiles correctly reconstruct the two peaks of the spatial distribution and the dip in between them, but vary by their intensity in a way which corresponds to the relative population of that wavelength in the beam. The profiles also vary in terms of the exact spatial coordinates they sample, this is because of the wavelength dependence of $k_{x}$ discussed earlier, which appears as sub-resolution shifts of the peak and dip positions.

To assess the usefulness of reconstructing wavelength resolved profiles, we have added to the plot the profile which is generated without resolving wavelengths (the black circular markers, which are identical to the purple traces shown in figure 1). For the case of a nominal reconstruction resolution of 50 μm (figure 1a), there is no significant advantage to reconstructing wavelength resolved profiles since the dip was clearly observable also in the profile generated without applying wavelength resolution. For the 10 μm data shown in figure 1b, the situation is very different. Without wavelength resolution the velocity spread we simulated (FWHM of 10%) completely obscured the dip, merging the two features into one wider peak, by contrast the wavelength resolved reconstructions (full lines following colour scheme shown in the legend) reconstruct the correct double peak profile successfully.

While the advantages of wavelength resolved imaging are clearly demonstrated for the higher resolution example, one potential problem which is expected when the technique is applied in an experiment is the expected loss of signal, as each wavelength resolved profile contains only a fraction of the signal that is created by the total beam. To overcome this, we can add the profiles of individual wavelength resolved reconstructions together to form the profile, shown as a dark blue line in figure 3. Note that first resolving the wavelengths and then adding the reconstructed profiles, is different than simply not resolving the wavelengths in the first place. This is clearly demonstrated when looking at the high resolution case (figure 3b) and comparing the sum of the wavelength resolved profile (dark blue line), which clearly shows the dip between two peaks, and the unresolved profile (black line), which loses that information due to velocity broadening in the beam.

Finally, it is important to note that combining the wavelength resolved profiles into one summed profile introduces some small (sub-resolution) broadenings. This is because the individual wavelength resolved profiles contain information at different spatial positions, and to combine them we had to first interpolate the profiles to a common spatial axis. For the profile shown in figure 3, and also later when we present experimental results, a simple first-order linear interpolation scheme is used.

## 5. An experimental measurement of $S\left(k_{x},k_{1}\right)$

The numerical simulations presented above clearly demonstrate that resolving wavelength can aid in mitigating velocity related broadening and improve image quality. To validate this technique experimentally and assess its feasibility, a transmission-mode experiment was conducted, closely following the imaging approach of the first MBSN demonstration [1], but with the addition that $B_{1}$ was varied to generate a 2D measurement of $S\left(k_{x},k_{1}\right)$.

**Figure 4.**
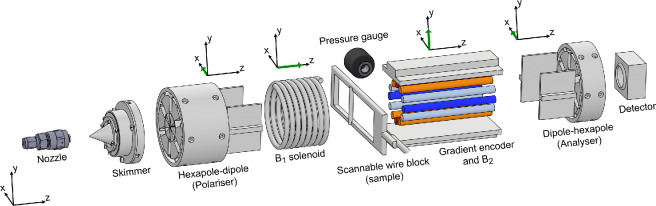
Schematic of experimental set-up. The molecular beam travels along $\hat{z}$ from left to right. A supersonic beam is created at the nozzle and collimated by a skimmer. The first magnetic element is a hexapole lens followed by a hexapole-to-dipole transition element where one spin projection is selected, focused to a parallel beam and aligned along the $\hat{x}$ axis. The beam traverses a homogeneous field produced by a solenoid, *B*_1_ before interacting with the sample (wire block). The beam then enters the encoding device (combination of the gradient field and the 2nd homogeneous field, *B*_2_). Finally, the beam passes through another pair of dipole–hexapole magnets which only allows particles with a positive spin projection along $\hat{x}$ to continue towards the detector. Further details of the experimental set-up can be found in [1].

Figure 4, reproduced from reference [1], illustrates schematically the set-up which was used to perform the first MBEM experiments, and also the measurements presented in this paper. We begin with a supersonic expansion through a 20 micron diameter nozzle and a subsequent collimation through a skimmer, to produce a continuous beam of parallel ^3^He atoms propagating along the $\hat{z}$-axis. The beam is initially state selected through a polarizing hexapole magnet [15] followed by a hexapole to dipole transition element which both focuses the beam to parallel and polarizes it, producing a magnetic moment projection which is aligned along $+\hat{x}$. Next, the beam traverses a homogeneous solenoid field, $B_{1}$, which we use in this study to scan the reciprocal variable $k_{1}$ and obtain wavelength resolution. The beam then continues towards the sample where the interaction with the sample modifies the spatial distribution of the beam which continues into the encoding element. To add some structure to the beam profile, a 100 μm wire was positioned vertically into the beamline, approximately 0.4 mm from the centre of the beam. The beam then enters an encoding element which produces a combination of a magnetic field gradient, $\frac{dB_{y}}{dx}$ and homogeneous holding field, $B_{2}$, also in the $\hat{y}$ direction, further explained below. Finally, the beam which exits the encoder continues towards another dipole–hexapole magnetic lens pair [16], which transmits atoms towards the detector chamber in a way which depends on the projection of their nuclear spin onto the $\hat{x}$ axis, forming the detected signal $S\left(k_{x}\right)$.

We now explain the various roles of the two homogeneous fields, $B_{1}$ and $B_{2}$. To produce the encoding field gradient, $\frac{dB_{y}}{dx}$, we used a 12-wire configuration of parallel current carrying conductors with a current distribution following $I=I_{0}cos\left(2\theta \right)$ [17]. While this configuration produces a highly linear and homogeneous gradient field, it also produces an additional orthogonal gradient field to that which we want to generate, i.e. the total field the device generates depends on both the $\hat{x}$ and $\hat{y}$ position $B_{encoding}=-y\frac{dB_{x}}{dy}+x\frac{dB_{y}}{dx}$. The presence of this additional gradient component contributes an additional spin-phase which would act to invalidate the encoding process. Our solution to this problem, was to super-impose a strong (an order of magnitude stronger) homogeneous field onto the gradient field, $B_{2}$, as a means of ensuring that precession is induced primarily in the desired xz plane for our measurement and little polarization is lost towards the $\hat{y}$ direction. However, the homogeneous field $B_{2}$ which solves the orthogonal gradient problem, simultaneously creates another problem, as the combination of a strong field and a velocity distribution with a non-negligible width leads to complete dephasing of the beam polarization. The $B_{1}$ field, which we used to scan $k_{1}$, also serves another role which is to counter the polarization dephasing in $B_{2}$. By setting the field integrals of the two homogeneous fields to have an equal magnitude, the dephasing taking place in $B_{2}$ can be reversed in $B_{1}$ leading to spin-echo refocusing [13,14]. Finally for completeness, we note that when the MBEM is used without wavelength resolved reconstruction, $B_{1}$ can be used to apply an additional $\frac{\pi }{2}$ current shift to measure the imaginary component of the signal, mimicking a measurement of two orthogonal spin projections [1]. In this study, scanning $B_{1}$ replaces the need to measure both the real and the imaginary components of $S\left(k_{x}\right)$. The range and increments of the gradient field were set to produce an FOV of 2.5mm and a scan resolution of approximately 50 μm. A total of 41 different $B_{1}$ excitations were applied, increasing the magnetic field path integral in steps of 0.056Gm, resulting in a relatively coarse resolution in wavelength space (i.e. 41 resolved wavelengths within the range −3.66 Å to +3.66 Å).

**Figure 5.**
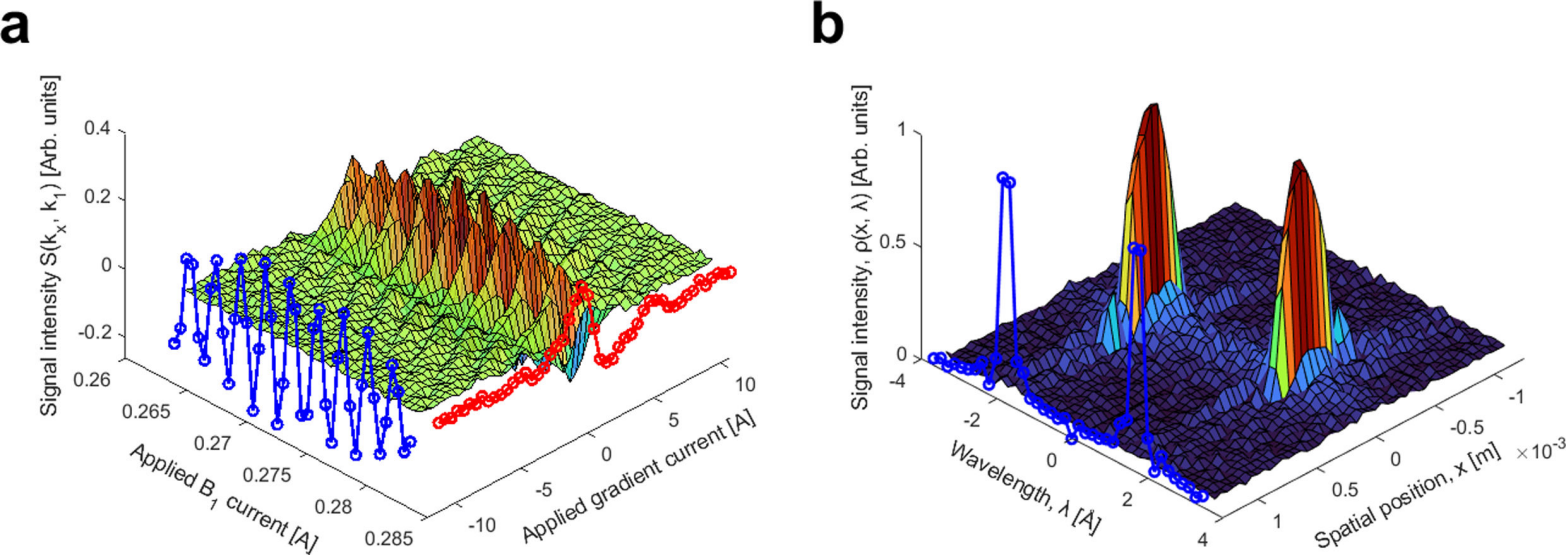
(a) Experimental raw signal *S*(*k*_*x*_,*k*_1_) for our beam distribution and beam energies. (b) Density array in position and wavelength, produced through two 1D Fourier transforms along each dimension, of the raw signal *S*(*k*_*x*_,*k*_1_).

The 2D signal $S\left(k_{x},k_{1}\right)$, presented in figure 5*a*, was produced by independently varying the current through both the gradient field and $B_{1}$ via the prescription outlined in steps 1 and 2 of figure 2. To assist the visualization of the 2D matrix $S\left(k_{x},k_{1}\right)$, two overlayed 1D curves are displayed. The blue curve shows the central region of the array when no gradient field is applied, showing the variation of $B_{1}$ around its spin-echo condition with $B_{2}$. The red overlayed curve was obtained by varying the gradient field strength while keeping $\left|B_{1}\right|=\left|B_{2}\right|$ and corresponds to the real component of $S\left(k_{x}\right)$ in the previous MBEM measurements [1]. Figure 5*b* shows the density array $\rho \left(x,\lambda \right)$ obtained by performing two sequential 1D Fourier transforms along each dimension of $S\left(k_{x},k_{1}\right)$. The blue overlayed plot in figure 5*b* shows the Fourier transform of a single row of the 2D matrix $S\left(k_{x}=0,k_{1}\right)$, which as can be seen by substituting $k_{x}=0$ equation (4.2), is equal to $\rho^{\prime }\left(\lambda \right)$, i.e. the distribution of wavelengths present within the molecular beam (mirrored for positive and negative wavelengths as discussed earlier).

**Figure 6.**
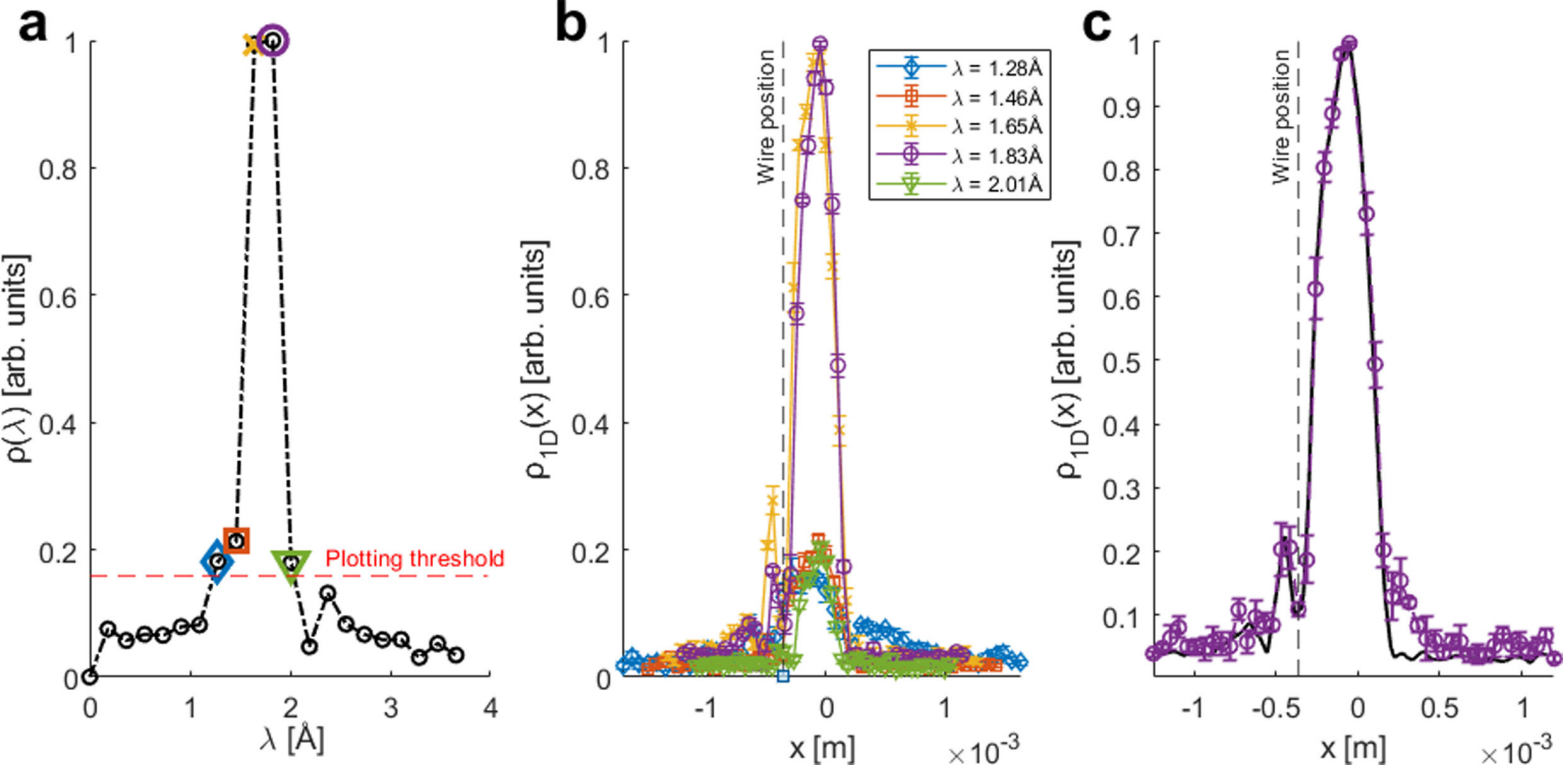
(a) Plot of wavelength intensities present in the beam. (b)Five highest intensity wavelength-resolved images along with the density of wavelengths within the beam signal. (c) Combined plot of the two highest intensity wavelength-resolved images interpolated and combined (black line), against the image produced with all velocities, i.e. without wavelength resolved imaging (circular purple markers).

Figure 6*a*, replots the reconstructed wavelength distribution obtained by Fourier transforming $S\left(k_{x}=0,k_{1}\right)$. From this distribution we chose the five wavelengths that were most prevalent in the molecular beam (highlighted with coloured symbols) and for these wavelengths we performed Fourier transforms along the $k_{x}$ dimension to obtain their wavelength resolved profiles presented in figure 6*b*. The reconstructed profiles which were produced from the two strongest intensity wavelengths (plotted using the orange x markers and purple circles) show the spatial profile we expected, i.e. the beam profile modulated by a sharp dip on the left side which was created by the wire positioned in the path of the beam, left of its centre. The other three profiles, which were reconstructed from the three wavelengths with significant less intensity, are noisier, but still follow the same basic pattern.

The black line in figure 6*c* shows a composite image produced by interpolating the two strongest wavelength resolved profiles shown in figure 6a to a common spatial axis and summing them. This procedure, as was demonstrated earlier using numerical simulations, can be used to compensate the reduction in the signal to noise ratio due to resolving the signal into its wavelength contributions. Finally we superimposed onto the same plot (figure 6c), a reconstructed profile which was obtained without applying the wavelength resolution scheme, plotted using purple circular markers. As was predicted from the numerical simulations, the two profiles in figure 6*c* are very similar. This is because for the relatively coarse spatial resolution of these experiments (50 μm) and a velocity distribution width of ≈7% FWHM in the helium beam we used, velocity broadening affects are expected to be negligible, as was demonstrated numerically in figures 1a,3 and 3a. Nevertheless, the comparison provides an experimental demonstration that the wavelength resolution scheme works as expected and can be implemented using the same apparatus which was used to perform the proof of principle MBEM measurements [1], changing only the measurement and interpretation protocols. Closer inspection of figure 6c, shows that the wavelength resolved profiles produce a sharper drop on the right side of the profile. A possible explanation for this discrepancy could be an artefact in the reconstruction without wavelength resolution. When reconstructing profiles without wavelength resolution, the real and imaginary components of the signal need to be measured to avoid symmetrization of the reconstructed profile [13]. The two components are measured in practice by adding a $\frac{\pi }{2}$ shift using the B_1_ field. Slight inaccuracies in the determination of this shift leads to intensity mirroring around the *x* = 0 and a distortion of the reconstructed profile, similarly to the occurrence of ghost peaks in inelastic spin echo measurements [13,14]. The wavelength resolution scheme presented in this work, does not require experimental determination of the $\frac{\pi }{2}$ shift, which means it both avoids these mirroring artefacts and is also capable of reconstructing the profile at the *x* = 0 position, an image feature previously not ascertainable due to a subtraction of the mean signal count-rate [1].

## 6. Summary

We presented an extension to the recently developed MBEM technique for performing neutral beam microscopy, which bypasses the resolution restrictions related to the existence of different velocities within the particle beam. The scheme we present uses a homogeneous magnetic field to produce de Broglie wavelength resolved profiles, which allow the observation of features which would be otherwise obscured due to the velocity broadening effect. The wavelength resolved profiles can also be summed to compensate for signal to noise losses associated with splitting the signal into its different wavelength components.

We used numerical simulations to demonstrate the scheme works and showed the powerfulness of wavelength resolved imaging for spatial resolutions where velocity broadening affects would become the limiting factor to resolving fine features. We have also shown that it is possible to implement the new imaging scheme using the same apparatus which was used to perform the first proof of principle MBEM measurements [1]. Within the experimental conditions the test could be conducted at (i.e. a resolution of 50 μm for a beam possessing a velocity distribution with an FWHM of approx. 7%), velocity broadening affects are quite subtle, and as anticipated by the numerical resolution the reconstructed image is not substantially different than that obtained without wavelength resolution. Nevertheless, these experiments confirm the simulated results and lay the groundwork for future refinements towards a dedicated and more powerful imaging apparatus. In particular, any future improvements to the spatial resolution of MBEM, which could be obtained by using a slower atomic beam, changing the length of the encoding element or strengthening the magnetic field gradient [1], would benefit significantly from implementing the wavelength resolved imaging scheme presented above.

## Data Availability

Data and relevant code for this research work are stored in GitHub [18] and have been archived within the Zenodo repository [19].

## Appendix A. Derivation of the dual transform

Here we provide more details about the analytical form of the measured experimental signal $S\left(k_{1},k_{x}\right)$ and the route taken to reconstruct wavelength-resolved images $\rho_{\lambda }\left(x\right)$; generated via independent field ramping of the homogeneous field $B_{1}$ and gradient field, $\frac{dB_{y}}{dx}$, which act to span both reciprocal spaces $k_{1}$ and $k_{x}$.

We rewrite equation (4.2) of the main text, but express $k_{x}$ explicitly to show its wavelength dependency:

(A 1)
$$S\left(k_{1},k_{x}\right)=\int_{-\infty }^{\infty }\rho \left(\lambda \right)e^{2\pi i\left(k_{1}\lambda \right)}\left\{\int_{-\infty }^{\infty }\rho \left(x\right)e^{i\gamma \frac{m\lambda L}{h}\frac{dB_{y}}{dx}x}dx\right\}d\lambda$$

Equation (A 1) can also be rewritten as

$$S\left(k_{1},k_{x}\right)=\int_{-\infty }^{\infty }f\left(\lambda ,k_{x}\right)e^{2\pi i\left(k_{1}\lambda \right)}d\lambda ,$$

if we define

(A 2)
$$f\left(\lambda ,k_{x}\right)=\rho \left(\lambda \right)\int_{-\infty }^{\infty }\rho \left(x\right)e^{i\gamma \frac{m\lambda L}{h}\frac{dB_{y}}{dx}x}dx.$$

This means that if we measure the signal as function of $k_{1}$ for a specific $k_{x}=k_{x}^{\prime }$, which we denote as $S\left(k_{1},k_{x}^{\prime }\right)$, we can perform an inverse Fourier transform to reconstruct $f\left(\lambda ,k_{x}^{\prime }\right)$.

(A 3)
$$f\left(\lambda ,k_{x}^{\prime }\right)=\int_{-\infty }^{\infty }S\left(k_{1},k_{x}^{\prime }\right)e^{-2\pi ik_{1}\lambda }dk_{1}$$

Following this procedure, we can obtain $f\left(\lambda ,k_{x}\right)$ for all values of λ and $k_{x}$. We now look at the definition of $f\left(\lambda ,k_{x}\right)$ in equation (A 2) which we have reconstructed, and note that for each individual $\lambda =\lambda^{\prime }$ it is essentially a Fourier transform of $\rho \left(x\right)$, where the value of $\lambda^{\prime }$ has two effects, one is rescaling the reciprocal variable $k_{x}$, and the second is a weighting factor $\rho \left(\lambda^{\prime }\right)$ multiplying the Fourier transform, which corresponds to how many particles in our beam have a wavelength of $\lambda^{\prime }$.

This means that for every $\lambda^{\prime }$ we can perform an inverse Fourier transform and extract the imaged profile for that wavelength,

(A 4)
$$\rho_{\lambda =\lambda^{\prime }}\left(x\right)\propto \int_{-\infty }^{\infty }f\left(\lambda^{\prime },k_{x}\right)e^{-2\pi ik_{x}x}dk_{x}$$

There are a few practical points to take into account. One is that the 2nd inverse Fourier step in (A 4) should only be performed for $\lambda^{\prime }$ values which are significantly populated within the beams (i.e. values where $\rho \left(\lambda^{\prime }\right)$) is large enough to contain useful information. A second point is that in practice we acquire a discrete and finite limit set and equations (A 1) to (A 4) should be replaced with their discrete Fourier transform equivalents, this leads to resolution and field of view values similarly to those discussed in the main text, where the only difference is that both the resolution and the field of view, which are dictated by the range and intervals of $k_{x}$, vary as function of the wavelength.
